# Development of Novel Rifampicin-Derived P-Glycoprotein Activators/Inducers. Synthesis, *In Silico* Analysis and Application in the RBE4 Cell Model, Using Paraquat as Substrate

**DOI:** 10.1371/journal.pone.0074425

**Published:** 2013-08-26

**Authors:** Vânia Vilas-Boas, Renata Silva, Andreia Palmeira, Emília Sousa, Luísa Maria Ferreira, Paula Sério Branco, Félix Carvalho, Maria de Lourdes Bastos, Fernando Remião

**Affiliations:** 1 REQUIMTE, Laboratório de Toxicologia, Departamento de Ciências Biológicas, Faculdade de Farmácia, Universidade do Porto, Porto, Portugal; 2 Departamento de Química, Laboratório de Química Orgânica e Farmacêutica, Faculdade de Farmácia, Universidade do Porto, Porto, Portugal; 3 Centro de Química Medicinal (CEQUIMED-UP), Universidade do Porto, Porto, Portugal; 4 REQUIMTE, Departamento de Química, Faculdade de Ciências e Tecnologia, FCT, Universidade Nova de Lisboa, Caparica, Portugal; University of Bologna & Italian Institute of Technology, Italy

## Abstract

P-glycoprotein (P-gp) is a 170 kDa transmembrane protein involved in the outward transport of many structurally unrelated substrates. P-gp activation/induction may function as an antidotal pathway to prevent the cytotoxicity of these substrates. In the present study we aimed at testing rifampicin (Rif) and three newly synthesized Rif derivatives (a mono-methoxylated derivative, MeORif, a peracetylated derivative, PerAcRif, and a reduced derivative, RedRif) to establish their ability to modulate P-gp expression and activity in a cellular model of the rat’s blood–brain barrier, the RBE4 cell line P-gp expression was assessed by western blot using C219 anti-P-gp antibody. P-gp function was evaluated by flow cytometry measuring the accumulation of rhodamine123. Whenever P-gp activation/induction ability was detected in a tested compound, its antidotal effect was further tested using paraquat as cytotoxicity model. Interactions between Rif or its derivatives and P-gp were also investigated by computational analysis. Rif led to a significant increase in P-gp expression at 72 h and RedRif significantly increased both P-gp expression and activity. No significant differences were observed for the other derivatives. Pre- or simultaneous treatment with RedRif protected cells against paraquat-induced cytotoxicity, an effect reverted by GF120918, a P-gp inhibitor, corroborating the observed P-gp activation ability. Interaction of RedRif with P-gp drug-binding pocket was consistent with an activation mechanism of action, which was confirmed with docking studies. Therefore, RedRif protection against paraquat-induced cytotoxicity in RBE4 cells, through P-gp activation/induction, suggests that it may be useful as an antidote for cytotoxic substrates of P-gp.

## Introduction

P-glycoprotein (P-gp) is a 170 kDa ATP-dependent transmembrane protein, belonging to the ATP binding cassette (ABC) superfamily, which promotes the outward transport of a wide spectrum of structurally unrelated compounds from various cell types [1]. It was firstly isolated from colchicine-resistant Chinese hamster ovary cells, where it modulated drug permeability [2], hence its name where P stands for “permeability”. P-gp has been initially associated to a multidrug resistance phenotype due to its overexpression in many cell types [3–8]. In fact, inhibition of its transport activity has long been seen as a strategy to overcome such resistance [9–12]. However, further studies suggested a protective role for P-gp (in alliance with metabolizing enzymes) due to its widespread constitutive expression in various blood-tissue barriers [13]. P-gp has been found physiologically expressed in enterocytes, hepatocytes and in proximal tubule cells in the kidneys [14], in the placenta and the testis [15] and also in the endothelial cells that compose the blood-brain barrier (BBB) [16]. The presence of P-gp at the BBB suggests an important role in protecting the brain against the noxious effects of P-gp substrates [8,17,18].

Given the importance of P-gp transport activity in the protection of sensitive tissues, such as the brain, P-gp activation/induction has previously been proposed as an antidotal way to prevent toxicity mediated by P-gp substrates such as paraquat (PQ) [19–21]. While a P-gp inducer promotes an increase in the transporter’s expression, from which is expected an increase in its activity, an activator is a compound that binds to P-gp and induces a conformational alteration that stimulates the transport of a substrate on another binding site. For example, Hoechst-33342 and Rhodamine-123 (Rho 123) act by this cooperative mode of action [22]. This functional model of P-gp suggested that the efflux pump contained at least two positively cooperative sites (H site and R site, for Hoechst-33342 and Rho 123, respectively) for drug binding and transport [22]. Therefore, this approach has the advantage of promoting P-gp transport function, without interfering with protein expression levels, which makes it a more rapid and clean process than P-gp induction. While some drug–drug interactions are still expected between P-gp activators/inducers and clinically used drugs that are substrates for P-gp (as occurs with P-gp inhibitors), these are expected to be attenuated, or even prevented, due to the short therapeutic period regularly required in an antidotal scheme.

**Figure 1:**
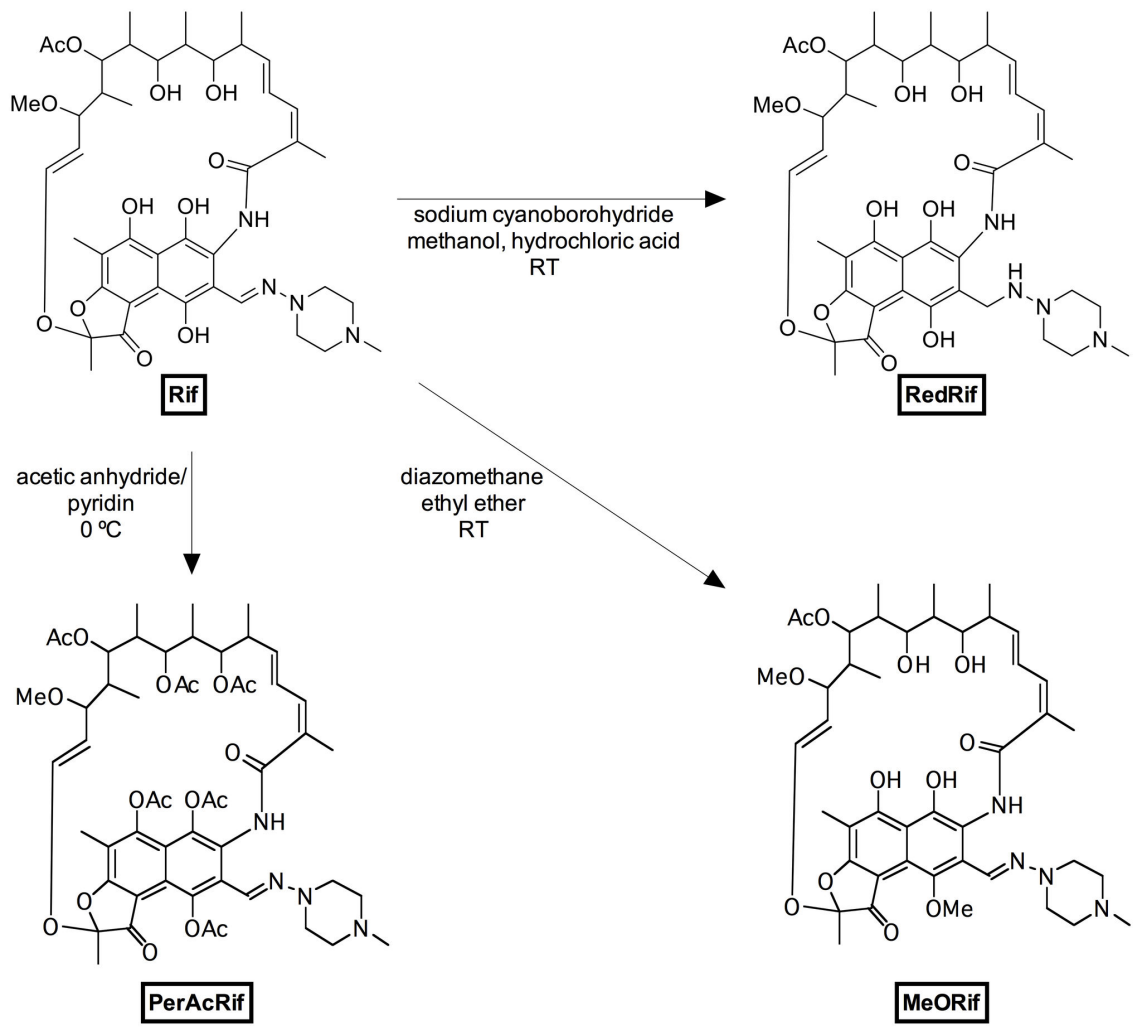
Reaction scheme for the synthesis of Rif’s derivatives. Rifampicin (Rif) was used as a model compound for the synthesis of three new derivatives on the search for new P-glycoprotein activators/inducers. A reduced derivative (RedRif), a peracetylated derivative (PerAcRif) and a mono-methoxylated derivative (MeORif) were obtained, as represented in the scheme. RT stands for room temperature.

Rifampicin (Rif, Figure 1) has been described to induce P-gp expression and activity in lymphocytes, intestinal cells and in renal cells, both *in vivo* and *in vitro* [23–26] via the pregnane-X-receptor (PXR) pathway. Although Rif’s ability to induce P-gp has been reported to be species-specific (due to ligand-binding cavity differences between human and rat PXR) some authors have recently reported Rif-induced P-gp overexpression *in vivo* in rat, and in rat cell lines and primary cultures [27,28]. In the present study we synthesized three Rif derivatives (a mono-methoxylated derivative – MeORif, a peracetylated derivative – PerAcRif, compounds that have never been described before, and a reduced derivative – RedRif, described for the first time on 2012 [29], Figure 1) in order to evaluate their ability to modulate P-gp expression and activity and also to determine their potential to protect against PQ-induced cytotoxicity, in an *in vitro* model of the BBB, the immortalized rat brain endothelial cell line, RBE4. This cell line expresses high levels of functional P-gp and is generally accepted as a suitable *in vitro* model for the study of transport functions of the BBB [30].

## Materials and Methods

### Chemicals and Materials

Minimum essential medium, nutrient mixture F-10 Ham, sodium bicarbonate, 4-(2-hydroxyethyl)-1-piperazineethanesulfonic acid (HEPES), neomycin sulfate, neutral red (NR) solution, ethyl alcohol absolute, acetic acid, methylthiazolyldiphenyl-tetrazolium bromide (MTT), dimethyl sulfoxide, monoclonal anti-α-tubulin antibody produced in mouse, rhodamine 123 (Rho 123), cyclosporin A (CyA), rifampicin (Rif) and paraquat dichloride (1,1′-dimethyl-4,4′-bipyridinium dichloride; methyl viologen dichloride hydrate - PQ) were all obtained from Sigma-Aldrich, Inc. (St. Louis, MO USA). Fetal bovine serum (FBS), antibiotic-antimycotic solution (10 000 U/mL penicillin G, 10 000 µg/mL streptomycin sulphate and 25 µg/mL amphotericin B in 0.85% saline), rat tail collagen type I, Hanks’ Balanced Salt Solution (either with or without Ca^2+^ and Mg^2+^ salts, +/+ or -/-, respectively), 0.25% trypsin-EDTA solution, were obtained from Gibco Laboratories (Lenexa, KS). Basic fibroblast growth factor (β-FGF) was from Invitrogen. MRP1 inhibitor, MK571, was obtained from Calbiochem (San Diego, CA). GF120918 was a generous gift from GlaxoSmithKline (Hertfordshire, United Kingdom). C219 anti-P-glycoprotein monoclonal antibody was obtained from Abcam. Secondary mouse antibody linked to horseradish peroxidase was from GE Healthcare Life Sciences. Bio-Rad DC protein assay kit was purchased from Bio-Rad Laboratories (Hercules, CA, USA). Flow cytometry reagents, Facs Flow (BD FacsFlow™) and *Facs Clean*, were obtained from Becton, Dickinson and Company, (San Jose, CA). Propidium Iodide was from BD Biosciences, Pharmingen (San Jose, California, USA).

All other reagents used were of analytical grade or of the highest available grade.

### Synthesis of rifampicin derivatives

Thin-layer chromatography (TLC) was conducted on Merck Kieselgel 60, F254 silica gel 0.2, 0.5 and 1 mm thick plates. Infrared (IR) spectra were recorded on a PerkinElmer Spectrum 1000 as potassium bromide (KBr) pellets. Proton and carbon nuclear magnetic resonance spectra (^1^H and ^13^C NMR) were recorded on a Bruker ARX 400 spectrometer at 400 and 100.62 MHz respectively. Chemical shifts are expressed in ppm, downfield from tetramethylsilane (δ = 0) as an internal standard; J-values are given in Hz. The exact attribution of NMR signals was performed using two dimension NMR experiments. The Fast Atom Bombardment (FAB) mass spectra were realized at University of Santiago Compostela, Spain (Unidade de Espectrometría de Masas).

Diazomethane was prepared by hydrolysis of an ethereal solution of *N*-methyl-*N*-nitroso-p-toluenesulfonamide (Diazald) according a well-established method [31].

The synthesized compounds were pure when analyzed by NMR. The elemental analysis of new Rif analogues RedRif and PerAcRif was carried out on a Thermo Finnigan Flash EA1112 (Bremen, Germany). The HPLC analysis of MeORif was conducted on a Merck, Hitachi system consisting of an L-7100 pump, a Rheodyne type injector, a D-7000 interface, and an L7450A diode array spectrometric detector, using a LiChrospher 100 RP-18 column, with water/methanol (at 2.5 pH) as mobile phase solvents.

### Reduced rifampicin (RedRif)

Rif (100 mg, 0.12 mmol) was dissolved in 10 mL methanol with a drop of HCl. The reductant sodium cyanoborohydride (NaBH _3_CN) was added portionwise (15.7 mg, 0.25 mmol) to the reaction mixture, at room temperature, until total consumption of Rif. The reaction mixture was evaporated to dryness, dissolved in 30 mL of methylene chloride and extracted twice with 30 mL of water and brine. The separated organic layer was evaporated and RedRif was purified by TLC in silica gel and methylene chloride/ methanol as eluent (10:1). Forty mg of an orange solid were obtained. Yield: 40%; m/z (FAB) [M+H]^+^ 825; IR (KBr, ν_max_ (cm^-1^)): 3390 (OH), 2937 (C–H), 1714, 1648 (C=O); ^1^H and ^13^C NMR similar to the ones described in literature [29]. Elemental analysis (%): calculated for C_43_H_60_N_4_O_12_:C, 62.66; H, 7.06; N, 6.43; found: C, 62.61; H, 7.33; N, 6.79.

### Peracetylated rifampicin (PerAcRif)

A solution of Rif (0.1 g, 0.12 mol) in acetic anhydride (1 mL) was added dropwise to a solution of pyridine (70 µL, 0.9 mmol) (0^°^C) in acetic anhydride (1.5 mL). The solution was stirred at 0^°^C until total consumption of Rif. The mixture was dropped over ice/water washed with methylene chloride. The organic phase was dried with anhydrous Na _2_SO_4_ and evaporated to dryness. The residue was purified by TLC and the product was obtained as a yellow solid: (49 mg, 0.05 mmol, η=39%), IR (KBr, ν_max_ (cm^-1^)): 2926 (C–H), 1780 and 1721 (C=O); ^1^H NMR (CDCl_3_, δ): 7.52 (1H, s, C_1’_–H), 6.05 (1H, t, J 12.0 Hz, C_18_H), 5.97 (1H, d, J 10.6 Hz, C_17_H), 5.51 (1H, m, C_19_H), 6.21 (1H, d, J 12.7 Hz, C_29_H), 5.21 (1H, dd, J 12.0, 6 Hz, C_28_H), 5.00 (1H, d, J 7.0 Hz, C_21_H), 4.89 (1H, d, J 10.0 Hz, C_23_H) 4.39 (1H, d, J 11.0 Hz, C_25_H), 3.18 (4H, m, C_4__’__,__8__’_H), 3.08 (1H, m, C_27_H), 3.03 (3H, s, C_37_H), 2.53 (4H, m, C_5__’__,__7__’_H), 2.50 (3H, s Ac1), 2.42 (3H, s Ac2), 2.34 (3H, s, C_9__´_H), 2.24 (6H, s, C_14_H, Ac3), 2.27 (3H, s, Ac1), 2.24 (6H, s, Ac2, Ac3), 2.00 (6H, s, Ac4, Ac5), 1.91 (3H, s, C_30_H), 1.88 (3H, s, C_36_H), 1.75 (3H, s, C_13_H), 1.51 (1H, m, C_26_H), 1.24 (3H, d, J 6.4 Hz, C_33_H), 0.91 (3H, d, J 7.0 Hz, C_31_H), 0.73 (3H, d, J 7.0 Hz, C_32_H), 0.49 (3H, m, C_34_H); ^13^C NMR (CDCl_3_, δ): 194.6 (C_11_), 174.5 (C_6_), 172.6 (OAc1), 170.8 (C_35_), 168.6 (C_15_ and =Ac2), 167.3 (C_8_ and OAc3), 167.0 (C_1_), 140.9 (C_29_), 135.5 (C_19_), 134.9 (C_16_), 130.1 (C_17_), 129.2 (C_18_), 126.6 (C_1’_), 120.8 (C_28_), 107.2 (C_12_), 77.3 (C_27_), 77.0 (C_25_), 76.1 (C_21_), 75.2 (C_23_), 57.3 (C_37_), 54.1 (C_5’7’_), 49.7 (C_4’8’_), 45.8 (C_9’_), 41.9 (C_20_), 39.5 (C_24_), 39.3 (C_26_), 35.2 (C_22_), 25.2 (Ac1), 20.9 (C_13,36_), 20.7 (Ac2-5), 18.8 (C_30_),18.5 (C_31_), 17.8 (C_32_), 9.0 (C_14_), 8.7 (C_33_), 7.5 (C_34_); it was impossible to identify the signal of C_5_, C_9_ e C_10_ on the spectrum; m/z (FAB) 1032.5 [M+1]. Elemental analysis (%): calculated for C_53_H_68_N_4_O_17_:C, 61.62; H, 6.63; N, 5.32; found: C, 61.65; H, 6.66; N, 5.10.

### Mono-methoxylated rifampicin (MeORif)

The ethereal solution of diazomethane was added to a solution of Rif (200 mg, 0.243 mmol) in ether at 0^°^C. After a reaction time of 3 h, the precipitate formed was removed, the solvent evaporated and the residue chromatographed by TLC in silica gel and methylene chloride/ methanol as eluent (10:1). The less polar component of the mixture was isolated as a red solid (6 mg, 0.0072 mmol, η=3%). IR (KBr, ν_max_ (cm^-1^)): 3444 (O–H), 2934 (C–H), 1736 and 1720 (C=O); ^1^H NMR (CDCl_3_, δ): 11.54 (1H, s, NH), 10.4 (1H, s, OH), 8.34 (1H,s,C _1_'H), 6.71 (1H, t, J Hz C_18_H), 6.41 (1H, d, J 11.2 Hz, C_17_H), 6.16 (1H, d, J 12.8 Hz C_29_H), 5.93 (1H, t, J 4.4 Hz, C_19_H), 5.1 (1H, t, J 6.4 Hz, C_28_H), 4.94 (1H, t, J 10.8 Hz C_25_H), 3.98 (3H, s, C_4´_-OMe), 3.44 (1H, m, C_23_H), 3.28 (2H, s, C_4,8__’_H), 3.06 (3H, s, C_37_H), 2.46 (2H, s, C_5__´__,__7__’_H), 2.34 (3H, s, C_14_H), 2.01 (3H, s, C_9__´_H), 2.07, 2.05 (2 x 3H, s, C_30_, C_36_H), 1.81 (3H, s, C_13_H), 0.87 (3H, d, J 6.8 Hz, C_32_H), 0.57 (3H, d, J 6.8 Hz, C_31_H), 0.07 (3H, d, J 6.8 Hz, C_33_H), -0.37 (3H, d, J 6.8 Hz, C_34_H); it was impossible to identify the signals for C_21_H, C_26_H and C_27_H; m/z (FAB) 1037,4 [M+1]. The purity of the compound was accessed by HPLC, as previously described (95%).

### Cell culture

Immortalized rat brain microvessel endothelial cells, RBE4 [32], were grown in minimum essential medium/Ham’s F10 (1:1) supplemented with 300 µg/mL neomycin, 10% FBS, 1 ng/mL β-FGF, 100 U/mL penicillin G, 0.25 µg/mL amphotericin B, 100 µg/mL streptomycin, 25 mM sodium bicarbonate and 25 mM HEPES. This mixture will herein be referred to as cell culture medium. These cells (passages 67-80) were maintained in a humidified atmosphere of 5% CO_2_ at 37^°^C. The cell culture medium was changed every 48 to 72 h. For sub-culturing, cells were dissociated with 0.25% trypsin-EDTA, which was neutralized with culture medium, and sub-cultured in 75 cm^2^ flasks. The RBE4 cells were, then, seeded on 6, 12 or 96-well collagen-coated plates.

### Cytotoxicity assays

Cells were seeded in 96-well plates at a density of 10 000 cells per well. Three days after seeding, cells were treated with 0.1 to 50 µM of Rif, MeORif, PerAcRif or RedRif, and cytotoxicity was evaluated after 24, 48 and 72 h by the NR uptake assay and by the MTT reduction assay. PQ cytotoxicity profile in this cell line has been previously established [33]. Each experiment was performed in triplicate and independently repeated at least 3 times.

### Neutral Red Uptake Assay

At the end of each predefined time-point, the cells were incubated with neutral red (50 µg/mL in cell culture medium, 90 min at 37°C). The dye absorbed by viable cells, was extracted (ethyl alcohol absolute/distilled water (1:1) with 5% acetic acid) and the absorbance was measured at 540 nm using a microtiter plate reader (PowerWaveX; Bio-Tek Instruments).

### MTT reduction assay

At the end of the incubation periods, 150 µL of 0.5 mg/mL MTT solution was added to each well, followed by incubation of the plates for 30 min at 37°C. The reaction was terminated by removal of the media and addition of 150 µL of dimethyl sulfoxide. Levels of reduced MTT were determined by measuring the absorbance at 550 nm using a microtiter plate reader (PowerWaveX; Bio-Tek Instruments).

### Western Blot analysis for P-gp expression assessment

Cells were seeded in 6-well plates at a density of 300 000 cells per well. Three days after seeding, cells were treated with 10 µM Rif, RedRif or PerAcRif or 5 µM MeORif. After 24, 48 or 72 h of incubation, cells were washed twice with HBSS (+/+) and lysed in a lysis buffer containing 1% Triton X-100, 5 mM ethyleneglycoltetraacetic acid, 150 mM NaCl in Tris-HCl 50 mM, pH 7.5, for 30 min at 4^°^C. Dithiothreitol 1 mM, phenylmethanesulfonylfluoride 0.25 mM and 1% of protease inhibitor cocktail (Sigma-Aldrich, Inc., St. Louis, MO, USA) were added to the buffer immediately before use. The lysates were centrifuged at 10 000 *g* for 10 min at 4^°^C and the supernatants were stored at −80^°^C until use. The protein content of each sample was determined according to Lowry’s method [34] using DC protein kit. The same amount of protein (35 µg) extracted from RBE4 cells was then separated by electrophoresis on a 7.5% SDS-polyacrylamide gel and electrophoretically transferred to a nitrocellulose membrane. The membrane was washed with Tris-buffered saline solution (TBS: 20 mM Tris-HCl, 300 mM NaCl, pH 8.0) and blocked in blocking buffer [TBS solution with 0.05% Tween-20 (TBS-T) and 5% dried skim milk], overnight, at 4^°^C. Then the membrane was incubated with the primary monoclonal antibody against P-gp, C219, diluted 1:400 in blocking buffer, overnight at 4^°^C or, in parallel, with anti-α-tubulin antibody (1:5000), to ascertain equal protein loading. After washing the membranes with TBS-T, these were incubated with secondary antibody (anti-mouse IgG-horseradish peroxidase, 1:1000 or 1:2000, respectively), at room temperature, for 3 h. Detection of protein bands was performed using ECL Plus chemiluminescence reagents (Amersham Pharmacia Biotech), according to the supplier’s instructions, and developed on high performance chemiluminescence films (Amersham Pharmacia Biotech) with Kodak Film Developer and Kodak Fixer (Sigma-Aldrich). Bands in the films were quantified using the ImageJ software (National Institutes of Health). Optical density results were expressed as percentage of control.

### P-gp activity assessment - Effects on Rho 123 accumulation

Cells were seeded in 12-well plates at a density of 200 000 cells per well. Three days after seeding, cells were exposed to the compounds for 24, 48 and 72 h. At the end of each time-point, cells were washed with HBSS (-/-), dissociated with 0.25% trypsin-EDTA and suspended in cell culture medium. Each collected well was divided into two aliquots. Half the aliquots were incubated with 2 µM Rho 123 in HBSS (+/+) supplemented with 10% FBS for 30 min, in the dark, in a shaking water bath, at 37^°^C. The other half was incubated in the same conditions but with 2 µM Rho 123 plus 10 µM CyA in HBSS (+/+) supplemented with 10% FBS. After this incubation period, cells were washed twice with ice-cold HBSS (+/+) and kept on ice until flow cytometry analysis. This assay was also performed replacing CyA for MK571, 20 µM, to depict any influence of MRP1 transporter in the efflux of Rho 123.

### Flow cytometry

Median fluorescence intensity values for each sample were assessed using a Becton Dickinson FACSCalibur™ flow cytometer (Becton Dickinson, Inc., Mountain View, CA, USA) equipped with a 488 nm argon-ion laser. Flow cytometry conditions were set as previously described [35]. Analysis was gated to exclude dead cells on the basis of their forward and side light scatters and the propidium iodide (5 µg/mL) incorporation, based on the acquisition of data for at least 10 000 cells. Obtained data were analysed using the BDIS CellQuest Pro software (Becton Dickinson, New Jersey, USA).

The green fluorescence due to Rho 123 was followed in channel 1 (FL1) and plotted as a histogram of FL1 staining. P-gp activity was expressed as percentage of control of Rho 123 efflux ratio, which was obtained by the ratio between median fluorescence intensity values of intracellular Rho 123 in the presence and in the absence of CyA.

### Effects on PQ-induced cytotoxicity

The effect of RedRif on PQ cytotoxicity profile was assessed either in pre-exposure to RedRif for 24, 48 and 72 h before PQ exposure for 48 h (P-gp induction effect), and in simultaneous exposure to RedRif and PQ for 48 h (P-gp activation effect). Briefly, three days after seeding in 96-well plates (10 000 cells per well), cells were exposed to 10 µM RedRif alone (pre-exposure) or simultaneously with growing concentrations of PQ (0.5-50 mM). For the pre-exposure assay, 24, 48 or 72 h after pre-exposure, RedRif was removed and replaced for growing concentrations of PQ. NR assay was performed as described above to assess PQ cytotoxicity 48 h after any exposure to PQ. A control for PQ cytotoxicity alone was performed in parallel in all procedures.

### P-gp’s role on RedRif’s protective effects against PQ-induced cytotoxicity

Cells were simultaneously exposed to 10 µM RedRif and growing concentrations of PQ (0.5-50 mM), in the presence or absence of 10 µM GF120918, for 48 h. NR uptake assay was then performed as described above.

### Docking on P-gp model

Docking simulations were done considering only the drug-binding pocket formed by the transmembrane domain interfaces of P-gp. Docking simulations between the P-gp model [previously described in [36]] and Rif, MeORif, PerAcRif, RedRif and 18 known P-gp activators [36–38] were undertaken in AutoDock Vina (Scripps Research Institute, USA). AutoDock Vina considered the target conformation as a rigid unit, while the ligands were allowed to be flexible and adaptable to the target. Vina searched for the lowest binding affinity conformations and returned nine different conformations for each ligand. AutoDock Vina was run using an exhaustiveness of 8 and a grid box with the dimensions 37.0, 30.0, 40.0, engulfing the channel formed by the transmembrane domains. Conformations and interactions were visualized using PyMOL version 1.3.

### Statistical analysis

All data are expressed as means ± standard deviation (SD). One-way analysis of variance (ANOVA) was used to determine the statistical significance of differences in cytotoxicity between control and each compound concentration. If analysis was significant, the differences were estimated using Dunn’s Multiple Comparison *post hoc* test. Two-way ANOVA followed by Bonferroni’s Multiple Comparison *post hoc* test was used to assess differences in P-gp expression and activity between control and treated cells throughout time, and to estimate RedRif’s effects on PQ cytotoxicity. The best-fit non-linear regression model was applied to evaluate differences between concentration–response curves to PQ-induced toxicity. The 0.05 level of probability was used as criterion of significance. All analyses were performed using GraphPad Prism software v 5.01 (GraphPad Software, San Diego, CA).

## Results

### Synthesis of RedRif, MeORif and PerAcRif

The synthesis of the three described compounds followed standard methods for the synthesis of reduction of imide, methylation of acidic hydroxyl groups and acetylation of hydroxyl groups. The structure of the used compounds was confirmed by two dimension NMR techniques (that allowed attributing the position of the new substituents on the Rif backbone) and mass spectrometry (that allowed confirming the number of new substituents). After our synthesis of RedRif other authors published the synthesis of the same compound by a similar method with identical results [29]. RedRif, MeORif and PerAcRif molecular structures and synthesis conditions are represented in Figure 1.

### Cytotoxicity profiles of Rif, RedRif, MeORif and PerAcRif

A concentration range of each compound (0.1 and 50 µM) was tested, in RBE4 cells, for incubation periods of 24, 48 and 72 h. In all cases, a viability rate of more than 85% was required to proceed the study. Cytotoxicity profiles for Rif, RedRif, PerAcRif and MeoRif are available as supplementary information (figures S1 and S2). Significant decreases in cell viability were observed at 50 µM Rif, RedRif and PerAcRif and 10 µM MeORif. Therefore, Rif, RedRif and PerAcRif were further tested in P-gp modulation studies at 10 µM and MeORif at 5 µM.

### Rif and RedRif increased P-gp expression in RBE4 cells

**Figure 2:**
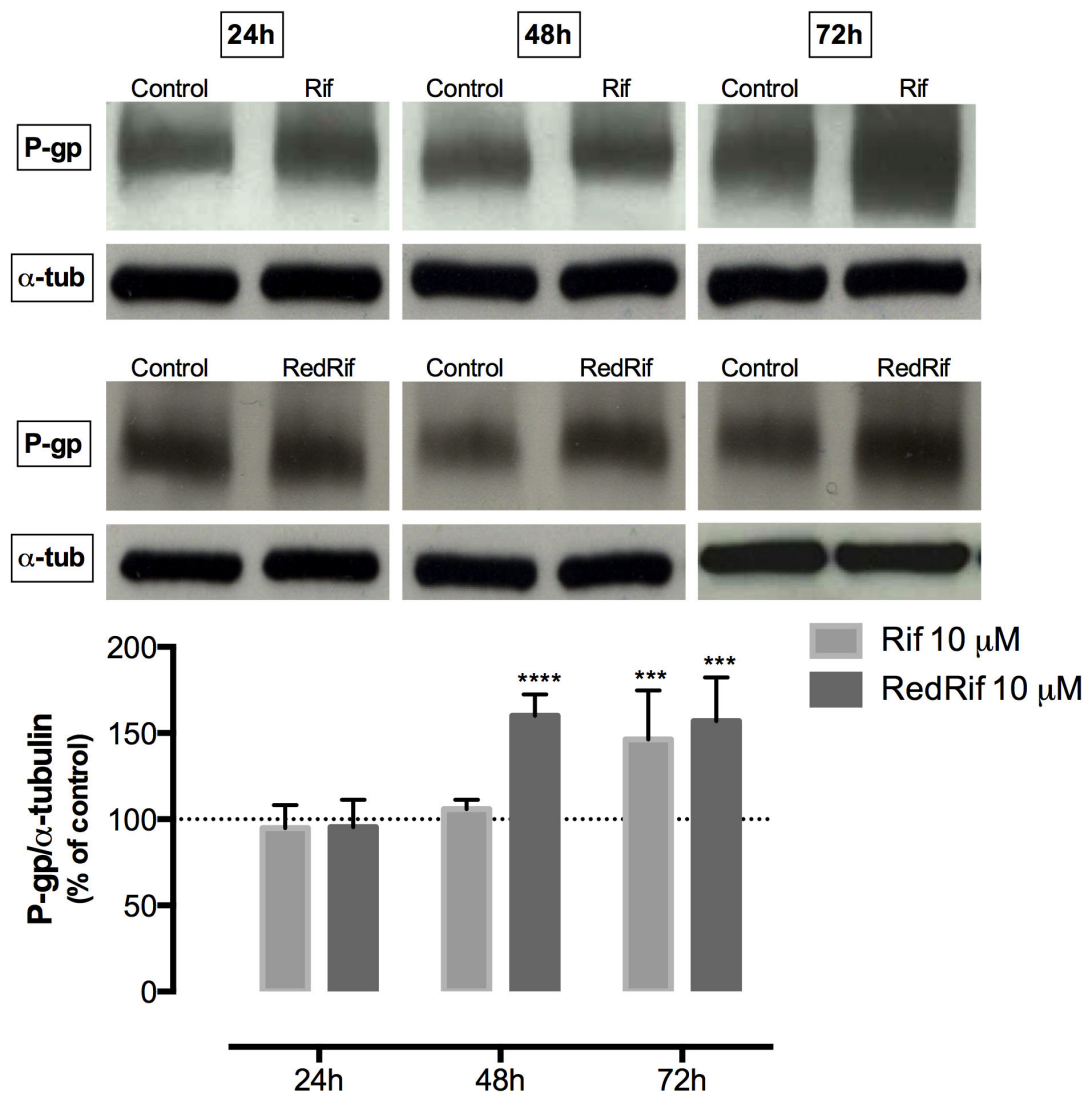
Rif and RedRif’s effect on P-glycoprotein expression. Cells were exposed to 10 µM Rif or RedRif and P-gp expression was evaluated by western blot after 24, 48 and 72 h of exposure, using C219 anti-P-gp antibody. Rif significantly increased P-gp expression after 72 h while RedRif induced a significant increase in P-gp expression from 48 h on. Results refer to mean ± SD of 3 or 4 independent experiments. Differences between treated and untreated cells were estimated using two-way ANOVA followed by Bonferroni’s multiple comparison *post hoc* test. ***p<0.001 and ****p<0.0001 *vs*. control.

P-gp expression assessment was performed by western blot using C219 anti-P-gp antibody. A significant increase in P-gp expression was found in Rif-treated cells after 72 h (p<0.001) and in RedRif-treated cells after 48 and 72 h of exposure (p<0.001), as shown in Figure 2. The remaining derivatives, MeORif and PerAcRif, did not significantly change P-gp expression in this cell line (data not shown).

### RedRif increased P-gp activity in RBE4 cells

P-gp function was evaluated by flow cytometry using a fluorescent P-gp substrate, Rho 123, in the presence and in the absence of P-gp inhibitor CyA, and represented as the ratio of Rho 123 transported out of cells. This is a widely used methodology to evaluate P-gp functionality in many cell types [20,35,39,40]. Although both Rho 123 and CyA have been reported to interact with multidrug resistance protein 1 (MRP-1) [41,42], and MRP-1 is known to be expressed in RBE4 cells [43], the contribution of this efflux pump to Rho 123 transport was negligible, as MK571 (a MRP-1 inhibitor) did not enhance the accumulation of Rho 123 in the cells (data not shown). This result indicated that Rho 123 and CyA are suitable tools to evaluate P-gp activity in this cell model, as previously suggested [44].

**Figure 3:**
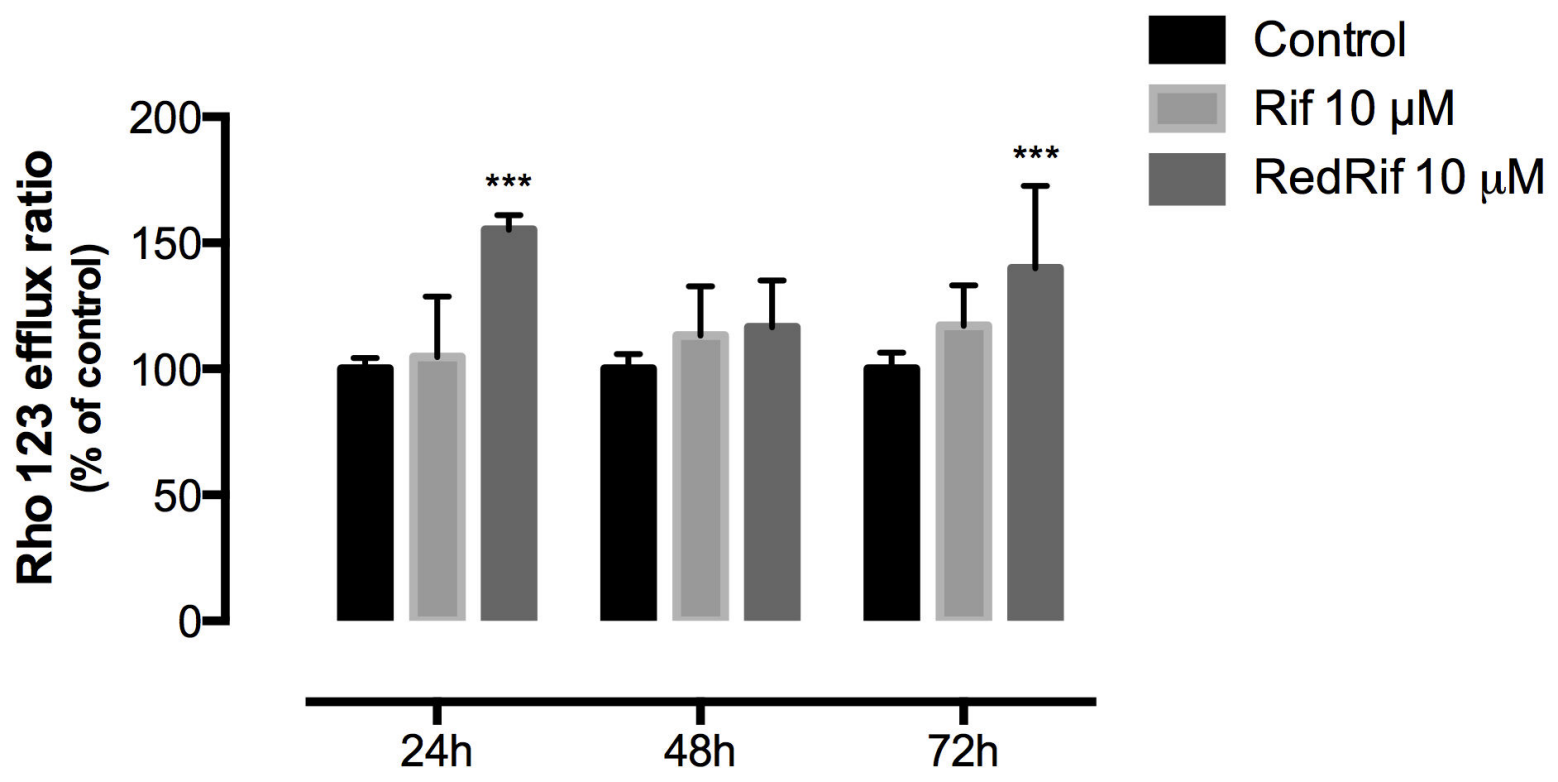
RedRif’s effect on P-glycoprotein activity – rhodamine 123 efflux ratio. P-gp activity is proportional to the ratio between Rho123 intracellular fluorescence from inhibited (+CyA) and non-inhibited (-CyA) cells. A significant increase in P-gp activity was found in RedRif-treated cells after 24 (P-gp activation effect) and 72h (P-gp induction effect) of exposure. Rif did not significantly change P-gp activity. Results refer to mean ± SD of at least 3 independent experiments performed in triplicate. Differences between treated and untreated cells were estimated using two-way ANOVA followed by Bonferroni’s multiple comparison *post hoc* test. ***p<0.001 *vs*. control.

RedRif induced a significant increase in Rho 123 efflux ratio after 24 and 72 h (p<0.001), as shown in Figure 3. The model compound, Rif (Figure 3), and the other derivatives (data not shown) did not significantly alter P-gp functionality in RBE4 cells.

### RedRif protects against PQ-induced cytotoxicity through P-gp activation

**Figure 4:**
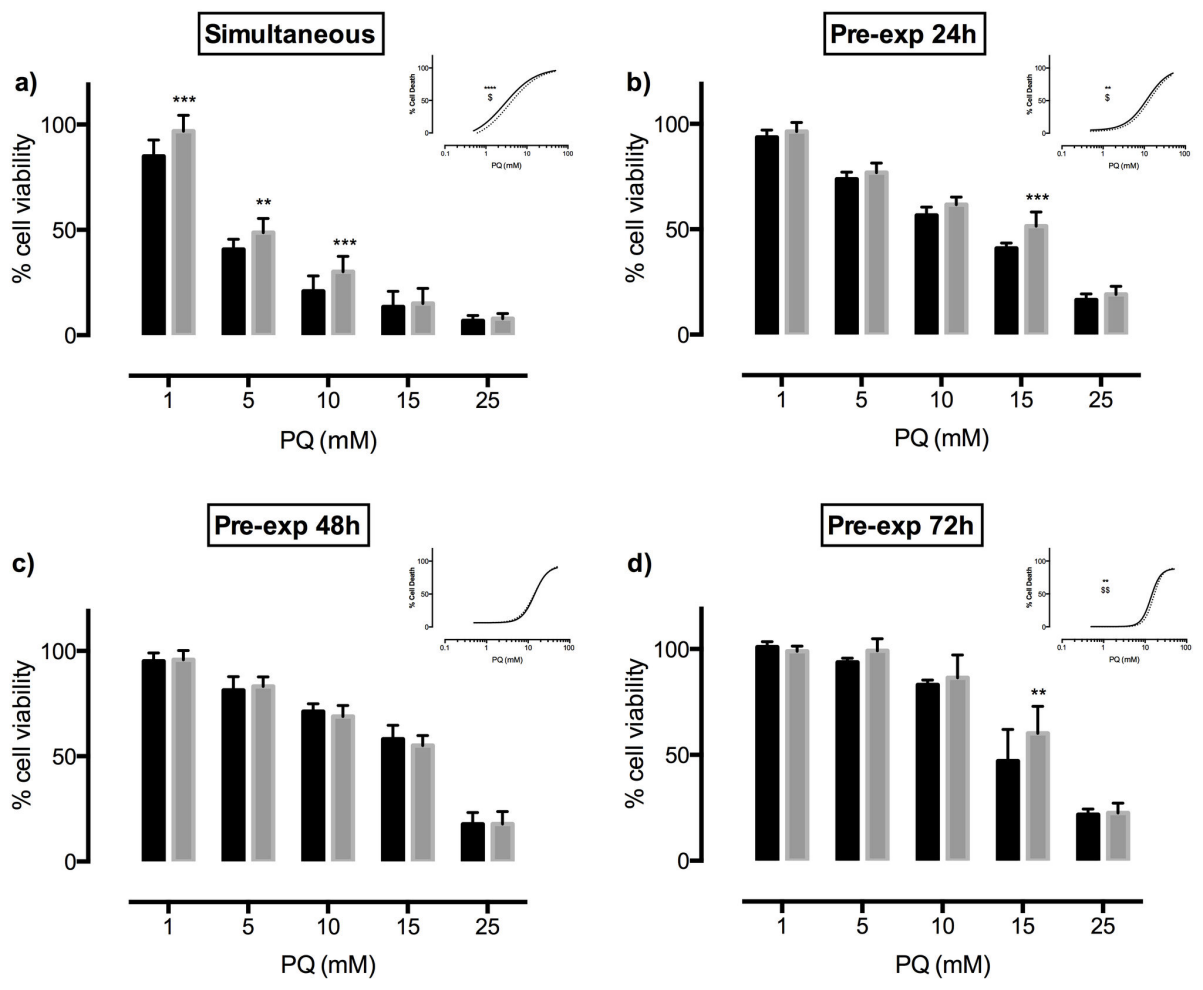
RedRif’s effect on paraquat-induced cytotoxicity. The NR uptake assay was performed to assess RedRif’s effect in PQ cytotoxicity in a **a**) simultaneous exposure to RedRif and PQ for 48 h – study of P-gp activation effect, and **b**) 24 h, **c**) 48 h and **d**) 72 h of exposure to RedRif before exposure to PQ – study of P-gp induction effect. RedRif’s protective effect against PQ-induced cytotoxicity was more significant in the simultaneous exposure assay. Two-way ANOVA was performed to estimate the differences between control (black bars) and RedRif-treated (grey bars) cells for each PQ concentration. Concentration–response curves shown as inserts were fitted using least squares as the fitting method, and the comparisons between the curves obtained in the presence and the absence of RedRif (bottom, top and EC_50_) were made using the extra sum-of-squares F test. At least 3 independent experiments were performed in triplicate. **p<0.01 and ***p<0.001 and ****p<0.0001 for differences between control and RedRif-treated cells for each PQ concentration and for differences between the curves *vs*. control; $p<0.05 and $ $p<0.01 for differences in EC_50_ *vs*. control.

The effect of the observed P-gp activation/induction by RedRif on the cytotoxicity profile of PQ in RBE4 cells was then evaluated. Simultaneous exposure to RedRif and PQ during 48 h significantly increased cell viability at 1 (p<0.001), 5 (p<0.01) and 10 (p<0.001) mM PQ-concentrations and resulted in significantly different curves (p<0.0001) and in a significant increase in PQ’s EC_50_ (p=0.0381), as demonstrated in Figure 4a. Pre-exposing cells to RedRif for 24 h led to a significant protection from PQ-induced toxic effect at 15 mM PQ (p<0.001), resulting in significantly different curves (p=0.0043), and increased PQ EC_50_ (p=0.0216), as represented in Figure 4b. This effect was also observed 72 h after exposure to RedRif (p<0.01 for 15 mM PQ; p=0.0003 for differences between the obtained curves; p=0.0006, for differences between EC_50_’s - Figure 4d).

**Figure 5:**
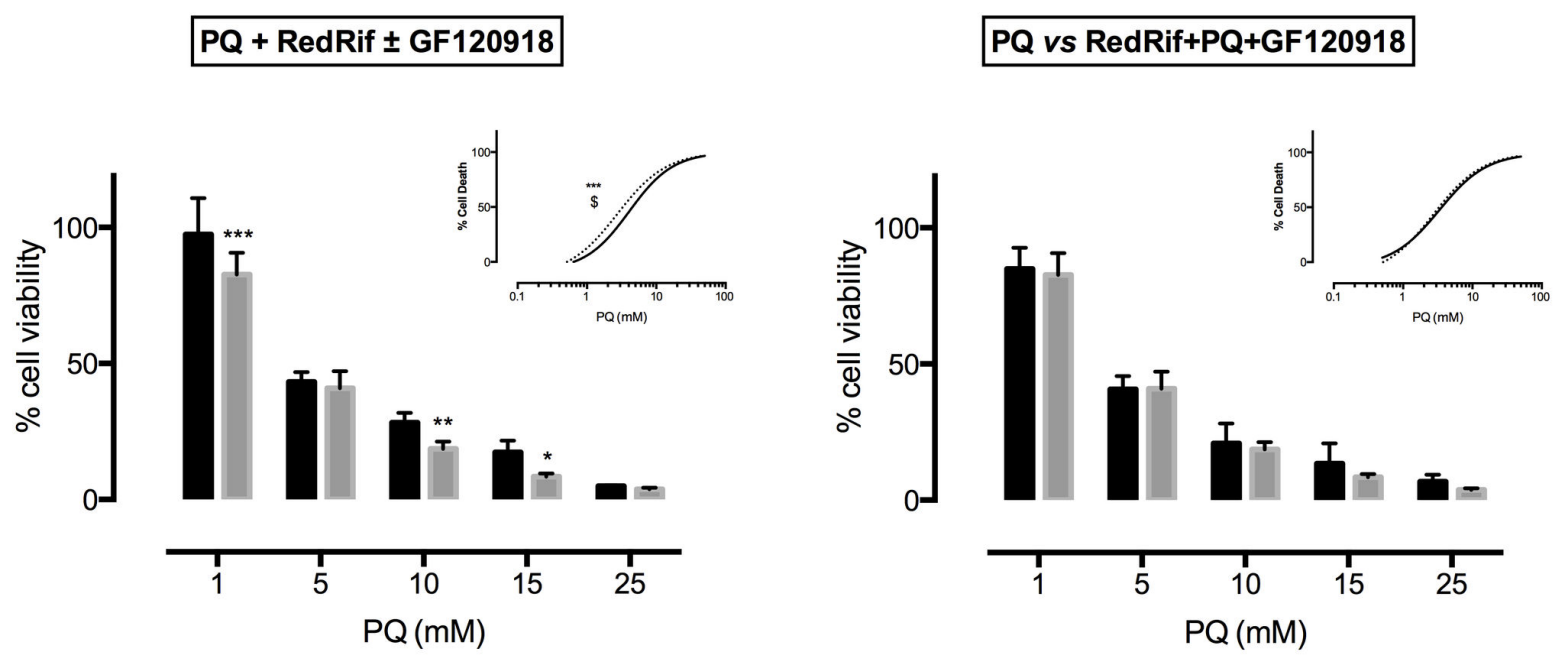
Reversal of RedRif-induced P-glycoprotein protective effect against paraquat cytotoxicity. **a**) Effect of P-gp blockade by potent P-gp inhibitor, GF120918, in cells simultaneously exposed for 48h to RedRif and PQ, with (black bars; dashed line) or without (grey bars; filled line) GF120918. **b**) All RedRif-induced protective effect was mediated by P-gp as no differences were observed between PQ-only and RedRif+PQ+GF120918-treated cells. Two-way ANOVA was performed to estimate the differences between RedRif+PQ or PQ (black bars) and RedRif+PQ+GF120918 treatment (grey bars) for each PQ concentration. Concentration–response curves were fitted using least squares as the fitting method, and the comparisons between the curves obtained in the presence and the absence of GF120918 (bottom, top and EC_50_) were made using the extra sum-of-squares F test. At least 3 independent experiments were performed in triplicate. Significant differences were observed in the presence of GF120918. *p<0.05, **p<0.01 and ***p<0.001 for differences related to the presence of GF120918 and for differences between the curves; $p<0.05 for differences in EC_50_ *vs*. control.

A similar assay was performed but including P-gp inhibitor, GF120918, to confirm the involvement of P-gp activation in RedRif’s protection against PQ cytotoxicity. The results are shown in Figure 5. Simultaneous exposure to RedRif and PQ with GF120918 resulted in a significant increase in PQ cytotoxicity (p<0.001 for 1 mM PQ; p<0.01 for 10 mM PQ and p<0.05 for 15 mM PQ), which led to significant differences between the obtained curves (p=0.0004) and to a significant decrease in PQ EC_50_ from 4.1 to 2.7 mM (p=0.0267). When comparing PQ-only to (RedRif+PQ+GF120918)-treated cells, no significant differences were observed at any studied PQ concentration. In fact, completely overlapping curves were obtained for both test conditions (Figure 5).

### RedRif fits in P-gp drug-binding pocket

**Figure 6:**
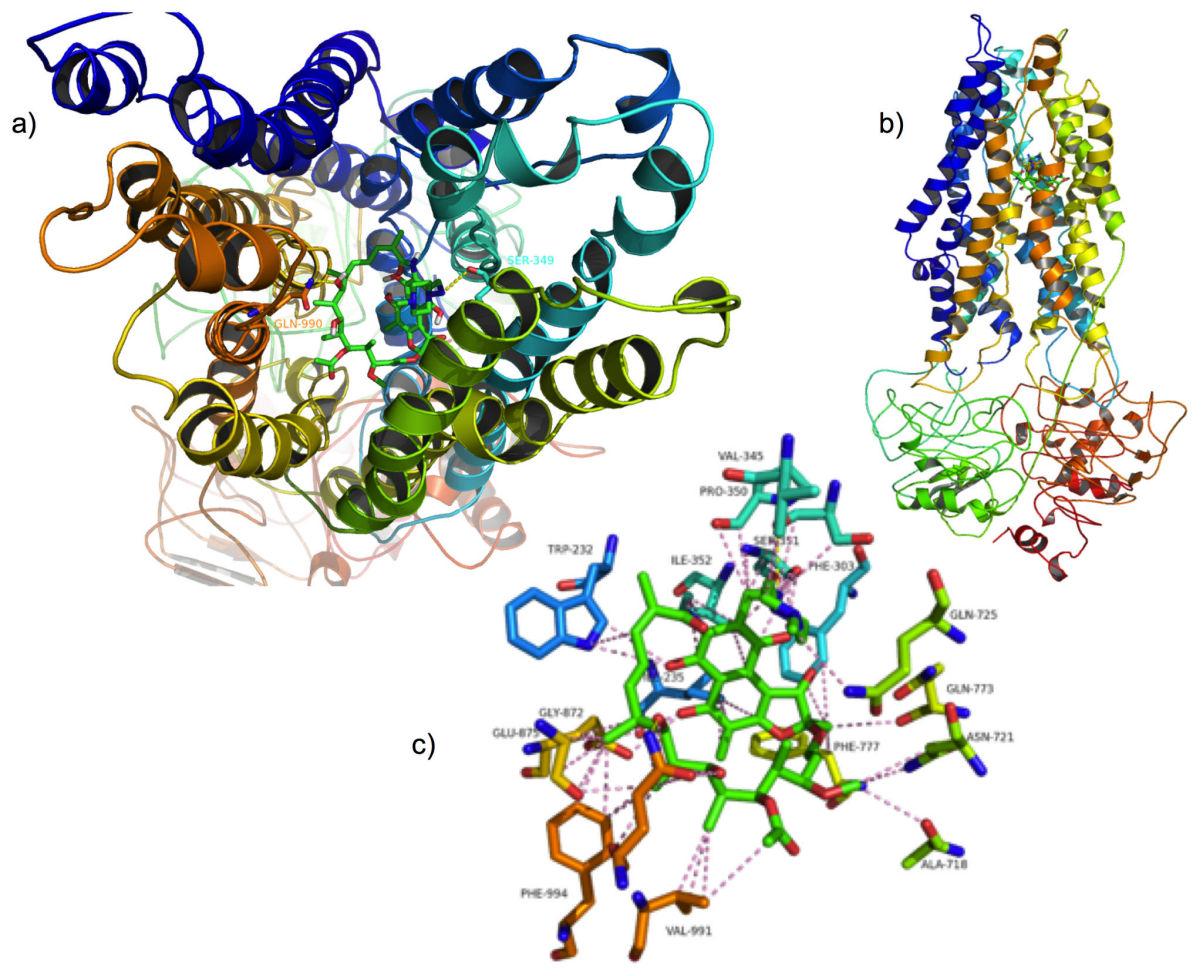
RedRif docked on P-glycoprotein. a) Top and b) side views. c) Detailed view of RedRif interactions with residues on P-gp. Hydrogen interactions are represented with yellow dashes. Other interactions are represented with pink dashes.

As P-gp activators bind in the drug-binding pocket formed by the transmembrane domain interface, a docking simulation of RedRif against P-gp was performed using a model built based on Sav1866, an ABC transporter from *S. aureus* [36]. Docking scores of nine RedRif conformations are described on Table 1 with the binding affinity value of the top rank conformation being -9.9 kJ.mol^-1^. Scores of known P-gp activators, used as controls, are available in Table S1. A visual inspection of the activator RedRif on the transmembrane domain was performed (Figure 6). Docking studies indicated that RedRif forms a more stable complex with P-gp than the other compounds and the known P-gp activators (lower free energy) as shown in Tables 1 and S1, respectively. Furthermore, RedRif has shape, size and stereoelectronic complementarity to P-gp drug-binding pocket (Figure 6), establishing hydrogen interactions with Serine-349 and Glutamine-990.

**Table 1:** RedRif, MeORif, PerAcRif, and Rif conformations rank and binding affinity (docking on P-gp model).

**Ligand**	**Conformation rank**	**Binding affinity (kJ/mol^-1^)**
**RedRif**	1	-9.9
	2	-9.7
	3	-9.3
	4	-9.1
	5	-9.1
	6	-9.1
	7	-9.0
	8	-8.9
	9	-8.9
**MeORif**	1	-8.4
	2	-8.3
	3	-8.2
	4	-8.1
	5	-8.1
	6	-8.1
	7	-8.0
	8	-8.0
	9	-7.9
**PerAcRif**	1	-7.2
	2	-6.8
	3	-6.7
	4	-6.4
	5	-6.3
	6	-6.2
	7	-6.1
	8	-6.0
	9	-6.0
**Rif**	1	-9.0
	2	-9.0
	3	-9.0
	4	-8.9
	5	-8.9
	6	-8.9
	7	-8.7
	8	-8.7
	9	-8.6

## Discussion and Conclusions

Rifampicin (Rif) is a bactericidal antibiotic that is mainly used to treat infections caused by *Mycobacterium* strains, like tuberculosis [45] and Hansen’s disease [46]. Prolonged therapy with Rif generally leads to therapeutic resistance due to activation of PXR, a master transcriptional regulator, which leads to induction of P-gp and CYP3A4 expression [47]. This P-gp-inducing ability of Rif has already been extensively reported [23–26]. Although Rif has been reported not to activate rat PXR, recent works have shown Rif-induced increases in rat’s P-gp, both at mRNA and protein levels [27,28]. On the basis of these facts, and due to our search for P-gp modulating agents, our synthesis group developed three new Rif derivatives, a mono-methoxylated derivative, MeORif, a peracetylated derivative, PerAcRif, and a reduced derivative, RedRif (Figure 1). These compounds are expected to follow the same metabolic pathway of deacetylation as the parent compound. A wide range of concentrations of these compounds was tested (0.1 to 50 µM), in RBE4 cells, in order to establish the concentration to be used in subsequent P-gp modulation studies. Their cytotoxicity profiles in RBE4 cells were fixed on the basis of MTT reduction and NR uptake assays. Rif, PerAcRif and RedRif were further tested at the concentration of 10 µM and MeORif at the concentration of 5 µM, as viability rates remained above 85% and no significant effects on cell viability were observed (Figures S1 and S2).

Western blot analysis revealed a significant increase in P-gp expression in Rif-treated cells 72 h after exposure without increasing P-gp functionality, which is in accordance with previous findings [28]. This may be explained by the fact that western blot was performed with whole cell lysates (membranes plus cytosol) and C219 anti-P-gp antibody recognizes an intracellular domain of P-gp. Therefore, total cell P-gp content was quantified, meaning that recognition of the protein does not necessarily indicate that it is integrated in the membrane or in its active form. Another explanation for this observation is the dual effect Rif is known to exert on P-gp, acting as an inducer in long-term exposures and also being a substrate for P-gp [48]. Therefore, despite inducing P-gp’s expression in our cell model, Rif may compete with Rho 123 to be transported through the efflux pump, thus diminishing the rate of transported Rho 123. Among the tested Rif derivatives, only RedRif led to a significant increase in P-gp expression after 48 and 72 h of contact with cells. Furthermore, RedRif significantly enhanced Rho 123 efflux ratio at 24 h, corresponding to an increase in P-gp activity. Once no P-gp expression increase was detected at this time-point, these results suggest that RedRif may act as an activator of P-gp efflux activity. On the other hand, at 48 h time-point, no changes in P-gp activity were observed despite the significant increases in P-gp expression. As already suggested for Rif, this may be due to the quantification of total P-gp content in RBE4 cells, suggesting that the protein is being actively synthesized but still may not be integrated in the membrane. After 72 h of exposure, the significant increase in P-gp activity observed in RedRif-treated cells was possibly due to the observed increase in P-gp expression at the same time-point. On the basis of these results, RedRif was the only compound to be tested against PQ-induced cytotoxicity.

Because PQ is a known P-gp substrate [19] with extensive documented toxic effects, an increase in the activity of this efflux transporter would decrease intracellular levels of PQ, consequently diminishing PQ-mediated cytotoxicity. RedRif’s ability to increase P-gp activity (and expression) was expected to produce such effect. To study P-gp induction effects, cells were exposed to RedRif for 24, 48 and 72 h before PQ exposure. A simultaneous exposure to RedRif and PQ for 48 h was also performed, to evaluate P-gp activation effects. We recently reported that RBE4 cells are highly resistant to PQ toxicity, which implies that all PQ exposures need to last 48 h, the time necessary for PQ to induce a significant toxic effect on RBE4 cells [33]. RedRif significantly protected RBE4 cells against PQ-induced cytotoxicity. This effect was much more significant when simultaneous exposure was performed than in pre-exposure assays, suggesting that P-gp activation by RedRif may be a more efficient way to prevent P-gp substrates’ toxicity.

In order to confirm P-gp’s involvement in RedRif-induced protective effect, the same assay was performed using RedRif and PQ in the presence and in the absence of P-gp inhibitor GF120918 in a simultaneous exposure. GF120918 significantly inhibited RedRif’s protective effect for the intermediate PQ concentrations (1, 10 and 15 mM). This effect resulted in a significant decrease in PQ EC_50_ from 4.1 to 2.7 mM, implicating P-gp on RedRif’s protective role against PQ cytotoxicity. In fact, the observed effect was totally mediated by P-gp as no differences were found between a control curve (PQ for 48 h) and the curve obtained in the simultaneous presence of RedRif, PQ and GF120918.

Although Shapiro has long ago reported the existence of at least two positively cooperative sites for drug binding and transport in P-gp [22,49], a four-P-gp-binding-sites model was more recently proposed, supporting the presence of three transport sites and one regulatory site. This last site allosterically alters the conformation of the transport binding sites from low to high affinity, increasing the rate of translocation for substrates [50]. Adaptation and survival mechanisms of living beings have allowed the binding of several xenobiotics at the same time to P-gp [51,52], increasing the transport of each other, not competing but activating the transportation cycle [53]. Therefore, the hypothesis of an activation mechanism of action for RedRif was further supported by a docking study. RedRif was docked on the cleft formed by the transmembrane alpha-helices of a P-gp model based on homologous *S. aureus* ABC transporter, Sav1866 [36]. A more stable complex was formed between RedRif and the used P-gp model than its analogues and the known P-gp activators (lower free energy), which suggest that RedRif may have higher affinity to P-gp binding site than these compounds (Tables 1 and S1). Also, RedRif’s shape, size and stereoelectronic complementarity to P-gp binding pocket, allows the establishment of hydrogen interactions with Serine-349 and Glutamine-990. This last residue has already been described as being part of the translocation pathway and being involved in the transport cycle [54]. These results indicate that RedRif has high probability of interacting with the translocation channel on P-gp, which supports the experimental data. In what concerns Rif and the other derivatives, a structure-activity relationship study revealed that peracetylation of Rif increases the steric impedance and changes the orientation of PerAcRif in the P-gp binding pocket. The CN double bond next to the piperazine ring (see Figure 1) rigidifies the molecule and sets torsion angles that do not benefit the establishment of interactions with the transporter. The binding affinity of the top rank conformation of PerAcRif to the efflux pump was higher than RedRif-P-gp complex (Δ=-2.7 kJ.mol^-1^, Table 1). The complex formed between the MeORif and P-gp model also has a higher free energy than RedRif-macromolecule complex (Δ=-1.5 kJ.mol^-1^, Table 1). Noteworthy, the pattern of polar interactions is also different, involving distinct residues (when comparing Figure S3 (middle) and Figure 6, respectively). Although Rif has a slightly higher binding affinity towards P-gp than RedRif (Table 1), the possibility of this compound being a substrate cannot be excluded, as suggested by the authors and by others [48]. On the other hand, the introduction of hydrophobic substituents to the positively charged drug is expected to furnish chemosensitizers, as described elsewhere [55].

In conclusion, RedRif is a new Rif derivative that protects RBE4 cells against PQ-induced cytotoxicity by increasing P-gp expression and activity, consequently leading to an enhancement of PQ efflux. RedRif’s activator effect on P-gp activity was confirmed by *in silico* studies and experimentally, and seems to be more effective than its induction ability. Therefore, RedRif should be further tested in other cell lines and *in vivo* to establish its use to efficiently prevent the toxicity of P-gp substrates.

## Supporting Information

Figure S1 **Rif and RedRif’s cytotoxicity profiles assessed by the MTT reduction and the Neutral Red uptake assays.** Cytotoxic effect was evaluated 24, 48 and 72 h after exposure to the compound in a concentration range between 0.1 and 50 µM. The compounds were non-cytotoxic until 10 µM. Results refer to mean ± SD of at least 3 independent experiments. Differences between concentrations were estimated using Kruskal-Wallis test (one-way ANOVA on ranks) followed by Dunn’s multiple comparison *post hoc* test. **p<0.01; ***p<0.001; ****p<0.0001 *vs*. control.
(TIFF)

Figure S2 **PerAcRif and MeORif’s cytotoxicity profiles assessed by the MTT reduction and the Neutral Red uptake assay.** Cytotoxic effect was evaluated 24, 48 and 72h after exposure to the compound in a concentration range between 0.1 and 50 µM. PerAcRif remained non-cytotoxic until 10 µM while MeORif started significantly diminishing cell viability at 5 µM. Results refer to mean ± SD of at least 3 independent experiments. Differences between concentrations were estimated using Kruskal-Wallis test (one-way ANOVA on ranks) followed by Dunn’s multiple comparison *post hoc* test. *p<0.05; **p<0.01; ***p<0.001; ****p<0.0001 *vs*. control.
(TIFF)

Figure S3 **PerAcRif (white), MeORif (magenta), and Rif (yellow) docked on P-glycoprotein.**
(TIFF)

Table S1 **P-gp activators described by the Rho 123 accumulation assay and respective docking scores (kJ) on transmembrane domains.**
(DOCX)
