# Supplementary figures and images for: Development of Novel Rifampicin-Derived P-Glycoprotein Activators/Inducers. Synthesis, In Silico Analysis and Application in the RBE4 Cell Model, Using Paraquat as Substrate
Source: PLoS One. 2013 Aug 26;8(8):e74425. doi: 10.1371/journal.pone.0074425 (PMC3753303; doi:10.1371/journal.pone.0074425)

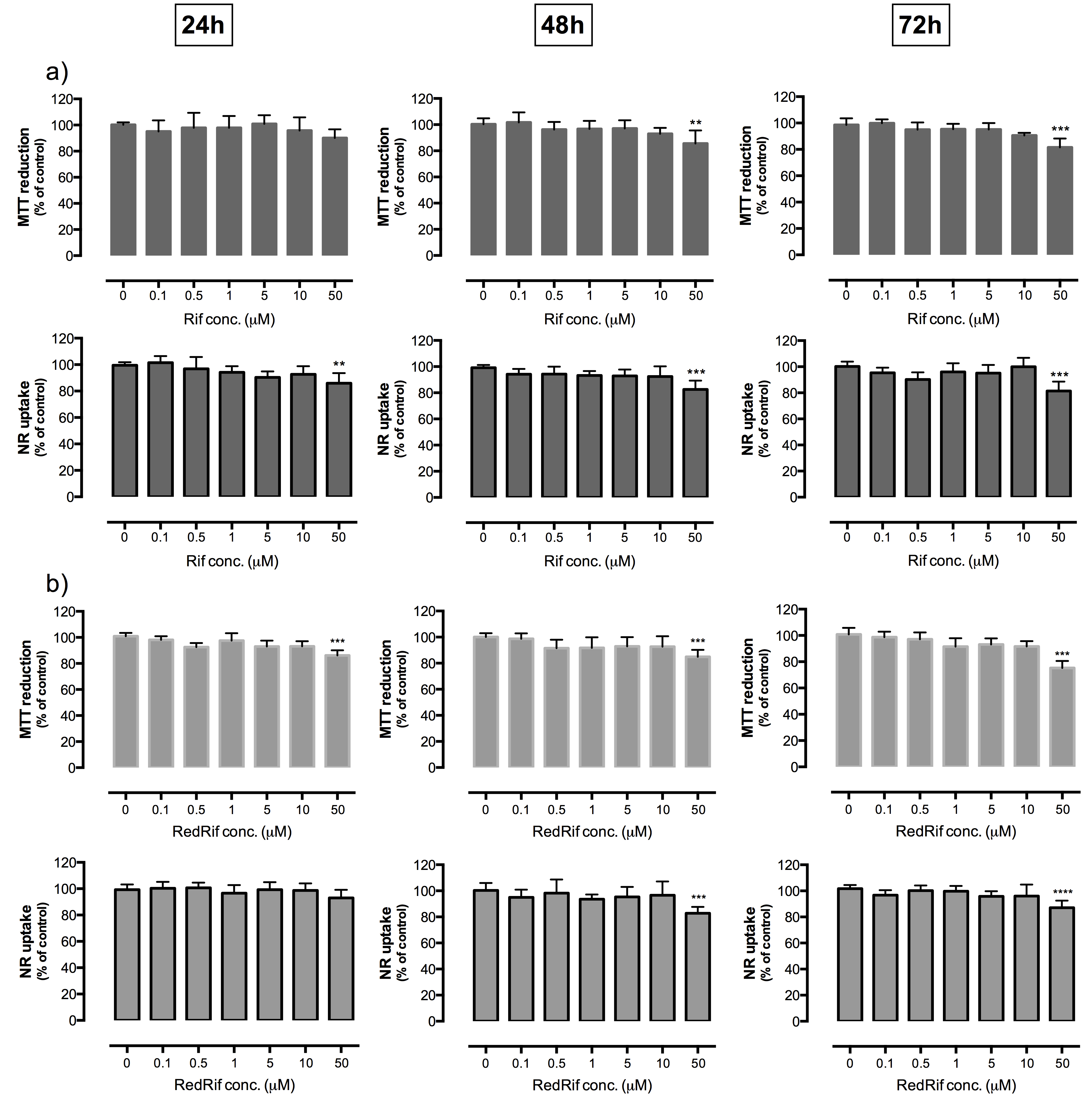

Supplement: Figure S1 — Rif and RedRif’s cytotoxicity profiles assessed by the MTT reduction and the Neutral Red uptake assays. Cytotoxic effect was evaluated 24, 48 and 72 h after exposure to the compound in a concentration range between 0.1 and 50 µM. The compounds were non-cytotoxic until 10 µM. Results refer to mean ± SD of at least 3 independent experiments. Differences between concentrations were estimated using Kruskal-Wallis test (one-way ANOVA on ranks) followed by Dunn’s multiple comparison post hoc test. **p<0.01; ***p<0.001; ****p<0.0001 vs. control. (TIFF) [file pone.0074425.s001.tiff]

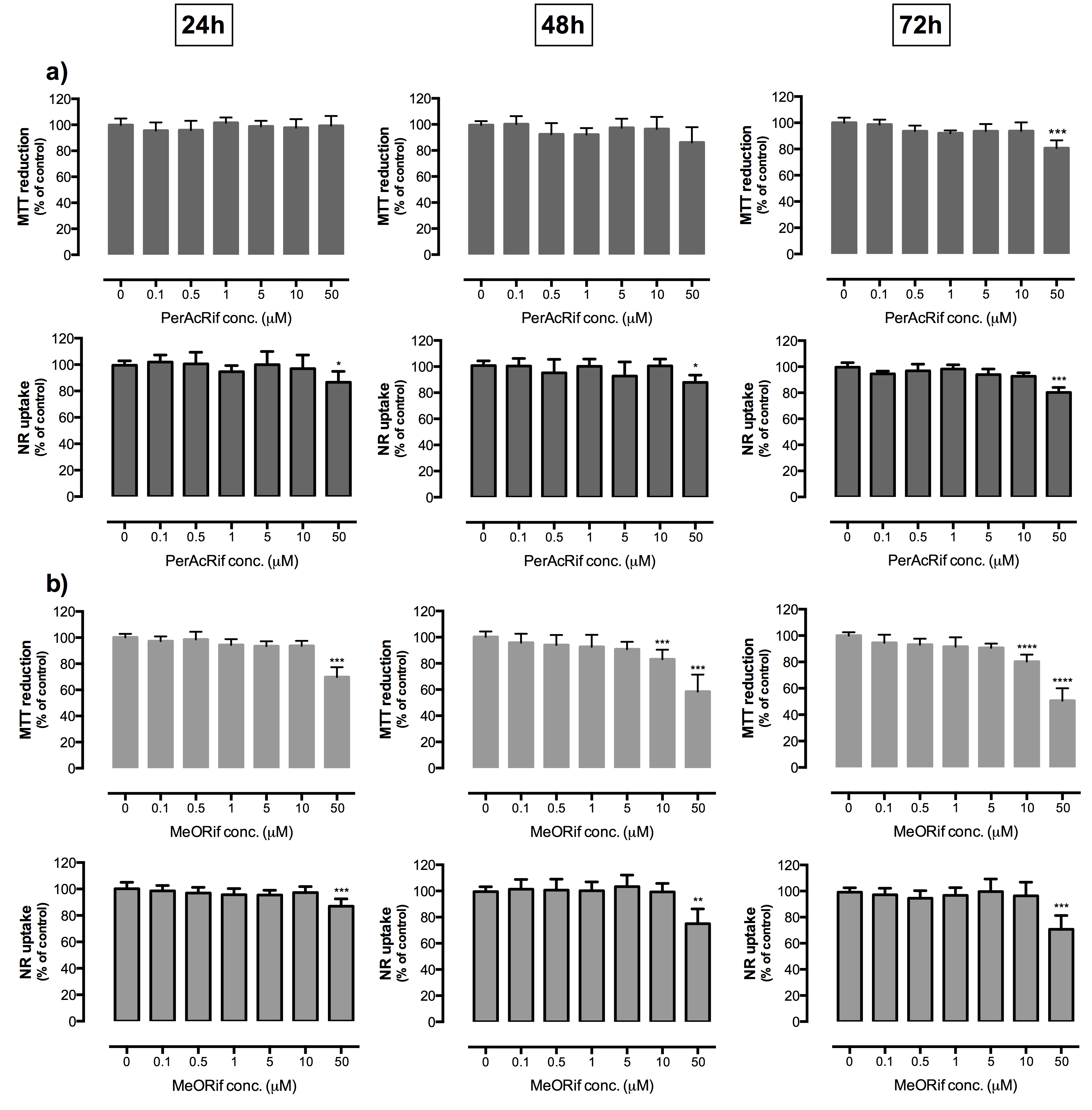

Supplement: Figure S2 — PerAcRif and MeORif’s cytotoxicity profiles assessed by the MTT reduction and the Neutral Red uptake assay. Cytotoxic effect was evaluated 24, 48 and 72h after exposure to the compound in a concentration range between 0.1 and 50 µM. PerAcRif remained non-cytotoxic until 10 µM while MeORif started significantly diminishing cell viability at 5 µM. Results refer to mean ± SD of at least 3 independent experiments. Differences between concentrations were estimated using Kruskal-Wallis test (one-way ANOVA on ranks) followed by Dunn’s multiple comparison post hoc test. *p<0.05; **p<0.01; ***p<0.001; ****p<0.0001 vs. control. (TIFF) [file pone.0074425.s002.tiff]

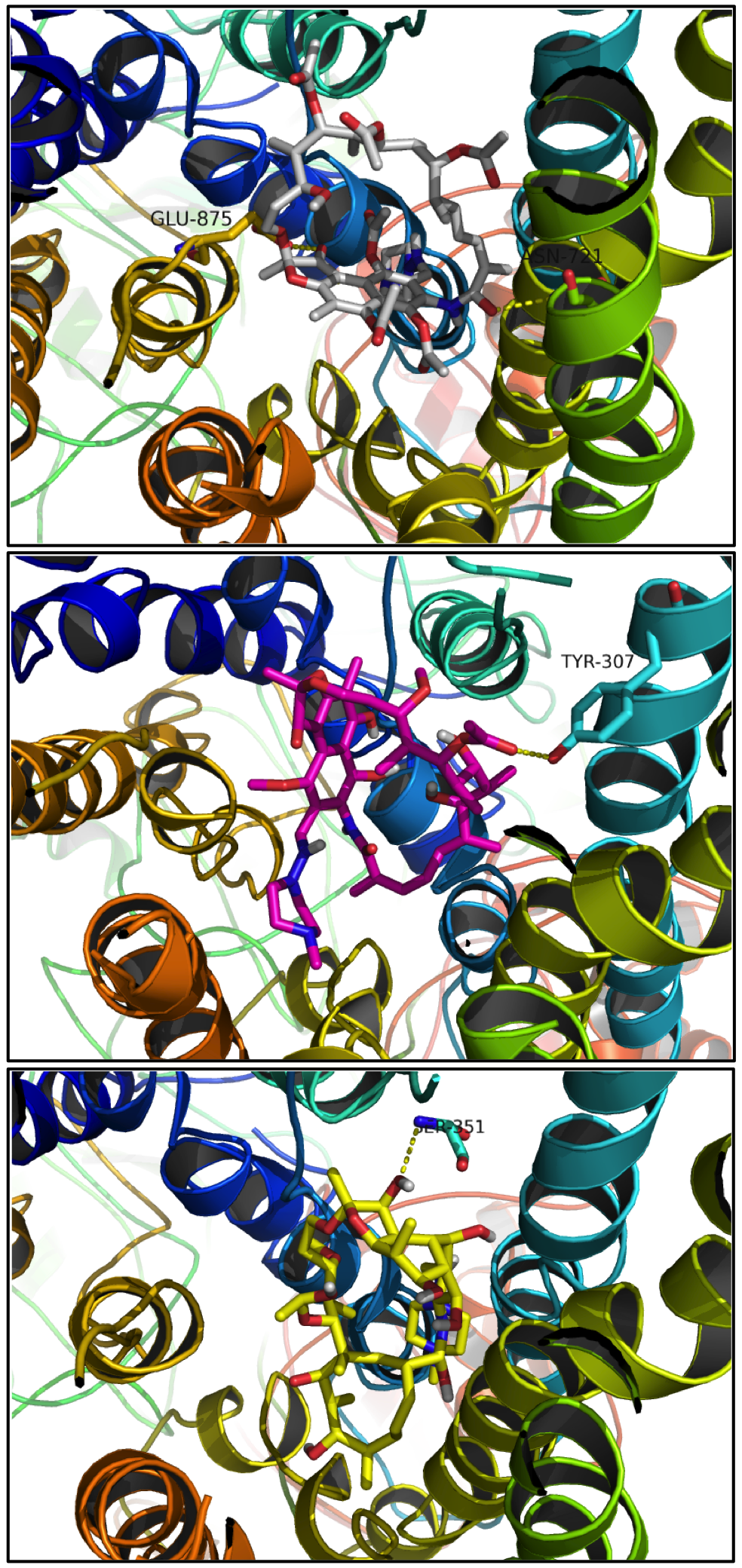

Supplement: Figure S3 — PerAcRif (white), MeORif (magenta), and Rif (yellow) docked on P-glycoprotein. (TIFF) [file pone.0074425.s003.tiff]
