# Supplementary material for: Development of Novel Rifampicin-Derived P-Glycoprotein Activators/Inducers. Synthesis, In Silico Analysis and Application in the RBE4 Cell Model, Using Paraquat as Substrate
Source: PLoS One. 2013 Aug 26;8(8):e74425. doi: 10.1371/journal.pone.0074425 (PMC3753303; doi:10.1371/journal.pone.0074425)
Supplement: Table S1 — P-gp activators described by the Rho 123 accumulation assay and respective docking scores (kJ) on transmembrane domains. (DOCX) [file pone.0074425.s004.docx]

**Table S1** – P-gp activators described by the Rho 123 accumulation assay and respective docking scores (kJ) on transmembrane domains

| Ligand | Binding affinity  (KJ.mol ^-1^) | Ref. |
| --- | --- | --- |
| 2-(4-methylphenyl)-5,6,7,8-tetrahydroimidazo[2,1-b][1,3]benzothiazole | -7.2 | [[1](#_ENREF_1)] |
| 2-phenyl-9-(prop-2-en-1-yl)-5,7,8,9-tetrahydro-6H-imidazo[1,2-a]benzimidazole | -7.6 |  |
| 1-{[4- (Aminomethyl)benzyl]amino}-4-propoxy-9H-thioxanthen-9- one | -7.8 | [[2](#_ENREF_2)] |
| 1-{[2- (Phenylamino)et hyl]amino}-4-propoxy-9H-thioxanthen-9-one | -7.5 |  |
| 1-[(3,4- Dimethoxybenzy l)amino]- 4-propoxy-9H-thioxanthen-9-one | -7.4 |  |
| 1-{[(2S)-1- Hydroxy-3- methylbutan-2-yl]amino}-4- propoxy-9H-thioxanthen-9-one | -7.2 |  |
| 1-[(3- Hydroxypropyl)a mino]-4-propoxy-9H-thioxanthen-9-one | -6.7 |  |
| 1-{[2-(1,3- Benzodioxol-5-yl)ethyl]amino}-4-propoxy-9H-thioxanthen-9-one | -8.1 |  |
| 1-[(2- methylpropyl)am ino]-4-propoxy-9H-thioxanthen-9-one | -7.5 |  |
| 1-(propan-2- ylamino)-4-propoxy-9H-thioxanthen-9- one | -6.5 |  |
| 1-chloro-9-oxo-9H-thioxanthen-4-yl acetate | -8.4 |  |
| 1-chloro-4-hydroxy-9H-thioxanthen-9-one | -6.8 |  |
| 4-hydroxy-9H-thioxanthen-9-one | -7.1 |  |
| 1,3-dihydroxy-9H-xanthen-9-one | -7.0 |  |
| Blebbistatin | -8.1 | [[3](#_ENREF_3)] |
| Coelenteramide | -7.2 |  |
| Indirubin | -6.5 |  |

1. Sterz K, Mollmann L, Jacobs A, Baumert D, Wiese M (2009) Activators of P-glycoprotein: Structure-activity relationships and investigation of their mode of action. ChemMedChem 4: 1897-1911.

2. Palmeira A, Vasconcelos MH, Paiva A, Fernandes MX, Pinto M, et al. (2012) Dual inhibitors of P-glycoprotein and tumor cell growth: (re)discovering thioxanthones. Biochem Pharmacol 83: 57-68.

3. Palmeira A, Rodrigues F, Sousa E, Pinto M, Vasconcelos MH, et al. (2011) New uses for old drugs: pharmacophore-based screening for the discovery of P-glycoprotein inhibitors. Chem Biol Drug Des 78: 57-72.
